# Cross-Sectional Study of Thiamine Deficiency and Its Associated Risks in Emergency Care

**DOI:** 10.5811/westjem.18472

**Published:** 2024-08-01

**Authors:** Joseph Miller, Daniel Grahf, Hashem Nassereddine, Jimmy Nehme, Jo-Ann Rammal, Jacob Ross, Kaitlin Rose, Daniel Hrabec, Sam Tirgari, Christopher Lewandowski

**Affiliations:** * Henry Ford Health and Michigan State Health Services, Department of Emergency Medicine, Detroit, Michigan; † Advocate Health, Division of Critical Care Medicine, Chicago, Illinois; ‡ Corewell Health University Hospital, Department of Emergency Medicine, Royal Oak, Michigan; § Henry Ford Health and Michigan State Health Sciences, Department of Internal Medicine, Detroit, Michigan; ∥ University of Michigan, Department of Emergency Medicine, Ann Arbor, Michigan

## Abstract

**Background:**

Growing data indicates that thiamine deficiency occurs during acute illness in the absence of alcohol use disorder. Our primary objective was to measure clinical factors associated with thiamine deficiency in patients with sepsis, diabetic ketoacidosis, and oncologic emergencies.

**Methods:**

This was an analysis of pooled data from cross-sectional studies that enrolled adult emergency department (ED) patients at a single academic center with suspected sepsis, diabetic ketoacidosis, and oncologic emergencies. We excluded patients who had known alcohol use disorder or who had received ED thiamine treatment prior to enrollment. Investigators collected whole blood thiamine levels in addition to demographics, clinical characteristics, and available biomarkers. We defined thiamine deficiency as a whole blood thiamine level below the normal reference range and modeled the adjusted association between this outcome and age.

**Results:**

There were 269 patients, of whom the average age was 57 years; 46% were female, and 80% were Black. Fifty-five (20.5%) patients had thiamine deficiency. In univariate analysis, age >60 years (odds ratio [OR] 2.5, 95% confidence interval [CI], 1.3–4.5), female gender (OR 1.9, 95% CI 1.0–3.4), leukopenia (OR 4.9, 95% CI 2.3–10.3), moderate anemia (OR 2.8, 95% CI 1.5–5.3), and hypoalbuminemia (OR 2.2, 95% CI 1.2–4.1) were associated with thiamine deficiency. In adjusted analysis, thiamine deficiency was significantly higher in females (OR 2.1, 95% CI 1.1–4.1), patients >60 years (OR 2.0, 95% CI 1.0–3.8), and patients with leukopenia (OR 5.1, 95% CI 2.3–11.3).

**Conclusion:**

In this analysis, thiamine deficiency was common and was associated with advanced age, female gender, and leukopenia.

Population Health Research Capsule What do we already know about this issue?

*Emergency patients with high metabolic strain, such as occurs in critical illness, have significant rates of thiamine deficiency in the absence of alcohol use disorder.*

What was the research question?

*Can clinical factors predict patients most likely to have thiamine deficiency in the absence of alcohol use disorder?*

What was the major finding of the study?

*Factors such as age >60 years (OR 2.0, 95% CI 1.0–3.8) and leukopenia (OR 5.1, 95% CI 2.3–11.3) are associated with deficiency.*

How does this improve population health?

*These findings point to the need for further investigation into micronutrient deficiency in populations that emergency clinicians commonly serve.*

## INTRODUCTION

Thiamine (vitamin B1) is a crucial cofactor for numerous metabolic processes, especially carbohydrate metabolism.^1^ Its deficiency is associated with diseases with significant morbidity, including Wernicke-Korsakoff syndrome and beriberi.^2^ Data in critical illness also shows an association with increased morbidity and mortality independent of these specific syndromes.^3^ ^,^ ^4^

Thiamine stores are entirely dependent on regular dietary intake or artificial supplementation, and body stores can become depleted within three weeks without adequate intake.^5^ ^–^ ^7^ Studies indicate that conditions routinely seen in the emergency department (ED) that increase metabolic strain are associated with thiamine deficiency, including diabetic ketoacidosis (DKA), sepsis, and cancer.^3^ ^,^ ^4^ ^,^ ^8^ ^–^ ^13^ In studies of severe sepsis and septic shock, up to 35% of patients show thiamine deficiency, with evidence that levels improve with recovery in children.^3^ ^,^ ^8^ ^,^ ^9^ ^,^ ^14^ Case reports of Wernicke’s encephalopathy in patients with cancer are numerous.^15^ ^–^ ^19^ In recent observational studies of gastrointestinal and hematological cancer patients, thiamine deficiency was common and associated with neurological symptoms.^20^ Studies have also demonstrated thiamine deficiency in up to 35% of children and adults with DKA.^10^ ^,^ ^11^

Although the existing literature demonstrates an association between such diagnoses and thiamine deficiency, further assessment of clinical risk factors for deficiency could define who may benefit most from thiamine treatment. In this study, we sought to explore the association of clinical risk factors with thiamine deficiency in ED patients. Such information could guide future therapeutic and preventative approaches in the management of at-risk patients.

## MATERIALS AND METHODS

### Design and Setting

This is an analysis of pooled data from two cross-sectional studies performed at a single-center, urban academic medical center. Enrollment occurred in the center’s ED, which has approximately 92,000 patient encounters each year and serves a primarily Black population. This research was approved by the institutional review board, which granted waiver of informed consent for the study.

### Population

This analysis was inclusive of adult patients (age ≥18 years) in the ED from two studies. The first study enrolled patients with diabetic or infectious emergencies from April 2015–February 2018. The second study enrolled patients with active malignancy between March 2017–October 2018. For both studies, we excluded patients who had known alcohol use disorder or received thiamine treatment in the ED or through home supplementation prior to enrollment.

### Blood Samples and Data Collection

Eligible patients provided a whole blood sample in the ED, which measures thiamine-diphosphate. The samples were protected from light, frozen, and sent to an offsite laboratory (Warde Medical Laboratory, Ann Arbor, MI) for thiamine-level testing using gas chromatography–mass spectrometry. We collected demographics, clinical characteristics, and in-hospital mortality data on all patients. We also recorded laboratory information obtained during routine care in the ED, including chemistries and blood counts. To account for missing laboratory data for serum albumin and hematology results, we performed multiple imputation prior to final analysis. Because preclinical data suggests that metformin may interfere with intestinal thiamine transporters, data specific to metformin use was also collected.^21^ Finally, we recorded any clinical documentation of abnormal gait or diagnosis of delirium throughout a patient’s ED or hospital stay. We did not perform standardized assessment of gait, delirium, or nutritional status.

### Statistical Analysis

We evaluated clinical characteristics and overall prevalence of thiamine deficiency using descriptive statistics. Discrete data was reported as frequencies and percentages. We reported continuous data as means or medians where appropriate. Analysis consisted of univariate comparisons and multivariable logistic regression to assess risk factors for the primary outcome of thiamine deficiency, defined as a whole blood level below the central lab’s reference range (38–122 micrograms per liter). Based on common clinically meaningful cutoffs, we created categorical variables for age >60 years (primary covariate of interest) and laboratory findings of leukopenia (white blood cell count <4 × 10^9^/L), moderate anemia (hemoglobin <10 grams per deciliter [g/dL]), and hypoalbuminemia (serum albumin <3.4 g/dL). With statistically significant categorical variables, we performed stepwise logistic regression (significance level for entry 0.2 and to stay 0.1) to construct a final model that was inclusive of age >60 years, gender, presence of leukopenia, and hypoalbuminemia. Analysis also included testing for an interaction of gender and age >60 years.

Results are reported as odds ratios (OR) with a 95% confidence interval (CI). A *P*-value <0.05 (two-tailed) was considered statistically significant for all tests. Due to the exploratory nature of this analysis, we did not perform a power analysis and used the sample size available. We used SAS 9.4 (SAS Institute, Inc, Cary, NC) for all analyses.

## RESULTS

There were 269 patients enrolled from March 2015–October 2018. Their mean age was 57 years, and 46% were female. Most patients identified as Black (80%). There were 55 (20.5%) patients who had thiamine deficiency. When compared to patients with normal thiamine levels, thiamine-deficient patients were older (*P* = 0.001) and more commonly female (*P* = 0.04). We report demographic and clinical characteristics with comparisons by thiamine status in Table 1.

**Table 1.** Demographics and clinical characteristics of patients based on presence or absence of thiamine deficiency.

	Normal thiamine N = 214	Thiamine deficiency N = 55	*P*-value
Demographics			
Female, no. (%)	91 (42.5)	32 (58.2)	0.04
Age, years, mean (SD)	54.8 (18.0)	63.6 (17.6)	0.001
Age >60 years, no. (%)	89 (41.6)	35 (63.6)	0.003
Black race, no. (%)	169 (79.0)	47(85.5)	0.28
Medical history, no. (%)			
Diabetes mellitus^*^	123 (57.5)	22 (40.0)	0.02
Metformin use	33 (15.4)	11 (20.0)	0.41
Myocardial infarction	20 (9.4)	2 (3.6)	0.17
Chronic kidney disease	57 (26.6)	16 (29.1)	0.72
Hypertension	131 (61.2)	35 (63.6)	0.74
Stroke	26 (12.2)	6 (10.9)	0.80
Cancer	74 (34.6)	29 (52.7)	0.01
Congestive heart failure	19 (8.9)	6 (10.9)	0.64
Laboratory values			
White blood cell × 10^9^/L, mean (SD)	13.5 (8.1)	8.1 (6.7)	<0.001
White blood cell <4.0 × 10^9^/L, no. (%)	18 (8.4)	17 (30.9)	<0.001
Hemoglobin, g/dL, mean (SD)	12.8 (3.2)	10.5 (2.7)	<0.001
Hemoglobin <10 g/dL, no. (%)	41 (19.2)	22 (40.0)	0.001
Creatinine, mg/dL, mean (SD)	2.20 (2.80)	1.64 (1.37)	0.04
Serum albumin, g/dL	3.43 (0.83)	2.86 (0.89)	<0.001
Albumin <3.4 g/dL, no. (%)	99 (46.3)	36 (65.5)	0.01
Body mass index, kg/m^2^, mean (SD)	27.2 (7.8)	27.6 (6.9)	0.75

* Diabetes mellitus included any patient with this clinical diagnosis (type 1 or 2). Regardless of being part of the diabetic ketoacidosis cohort.

*g/dL*, grams per deciliter; *mg*, milligrams; *kg/m* ^2^, kilograms per square meter.

Significant laboratory findings associated with thiamine deficiency in the cohort included leukopenia, anemia, and hypoalbuminemia. In the adjusted analysis, female gender, leukopenia, and age >60 years were significantly associated with thiamine deficiency. Table 2 demonstrates these unadjusted and adjusted associations. Finally, unadjusted rates of in-hospital mortality, 60-day mortality, findings of abnormal gait, and delirium in patients with or without thiamine deficiency are shown in Table 3.

**Table 2.** Unadjusted and adjusted analysis of patient characteristics and laboratory values associated with thiamine deficiency.

	Unadjusted analysis^*^		Adjusted analysis	
	Odds ratio (95% CI)	*P*-value	Odds ratio (95% CI)	*P*-value
Female gender	1.88 (1.03–3.43)	0.039	2.13 (1.11–4.07)	0.02
Age >60 years	2.46 (1.33–4.54)	0.004	1.98 (1.03–3.81)	0.04
Cancer	2.11 (1.16–3.84)	0.015		
Diabetes mellitus	2.03 (1.11–3.71)	0.022		
WBC <4^*^10^9^/L	4.87 (2.31–10.30)	<0.001	5.12 (2.31–11.30)	<0.001
Albumin <3.4 g/dL	2.20 (1.19–4.08)	0.012	1.82 (0.94–3.54)	0.08
Hemoglobin <10 g/dL	2.81 (1.49–5.32)	0.002		

* Categorical variables included in stepwise logistic regression model. Adjusted analysis displays only results for retained variables.

*CI*, confidence interval; *WBC*, white blood cell count.

**Table 3.** Unadjusted clinical outcomes based on presence or absence of thiamine deficiency.

	Normal thiamine N = 214	Thiamine deficiency N = 55	*P*-value
60-day mortality, no. (%)	34 (15.9)	15 (27.3)	0.05
Abnormal gait, no. (%)	58 (27.1)	25 (45.5)	0.009
Delirium, no. (%)	64 (30.1)	22 (40.0)	0.16

## DISCUSSION

It is well established that thiamine deficiency can result from alcohol use disorder or severe nutritional deficiencies. However, thiamine deficiency is also precipitated by acute illness with high metabolic demand, and data indicates it may be common in conditions such as DKA, sepsis, and cancer. Determining additional clinical factors associated with thiamine deficiency in such populations could aid in tailoring thiamine administration to appropriate patients.

Within this analysis of ED patients with sepsis, DKA, and cancer, we found that 20.5% of patients were thiamine deficient based on ED blood levels. We identified advanced age, female gender, and leukopenia as having greater adjusted odds of thiamine deficiency. These results on gender and leukopenia are unique. Prior data indicates an association between advanced age and thiamine deficiency.^22^ Gender or the presence of leukopenia are not well-described risk factors. Prior research indicates that B12 deficiency is more common in men,^23^ but we are not aware of data indicating a higher risk of thiamine deficiency based on female gender. The finding that hypoalbuminemia may be associated with thiamine deficiency is also novel, although this finding did not reach statistical significance in an adjusted analysis. It is also noteworthy that this analysis included mostly Black patients. Much of the existing literature on thiamine deficiency in critical illness is inclusive of a mainly White population.^3^ ^,^ ^10^ ^,^ ^20^ ^,^ ^22^ Nonetheless, we found overall comparable rates of thiamine deficiency within a Black cohort.

Nutritional deficiency may be contributory to our findings. While we did not perform nutritional surveys in these cohorts, other research indicates that ED patients have significant rates of malnutrition, approaching as high as 60% in the elderly.^24^ Future research in this area could benefit from specific measures of food insecurity and nutritional status in diverse populations. While we could presume that low albumin levels are reflective of poor nutritional status, it is challenging to determine whether hypoalbuminemia is due to poor protein intake vs inflammatory conditions or high metabolic demands that suppress albumin synthesis.^23^

## LIMITATIONS

The current study has several notable limitations. First, we included a narrow subset of diagnoses, which limits the generalizability of these findings to other conditions. Overall patient enrollment was low due to limited coordinator coverage, which may have introduced bias in patient selection. As noted, we did not perform standardized nutritional assessments or neurological assessments that might have teased out the clinical impact of thiamine deficiency. Of note, our results highlighted detection of acute deficiency and did not measure global thiamine stores. We performed this in an urban setting with significant rates of food insecurity. Such findings may not translate to suburban settings.

The study was also underpowered to assess certain drugs associated with thiamine deficiency such as furosemide.^25^ Nonetheless, these results add to a growing body of evidence on thiamine deficiency in ED patients with conditions such as DKA, sepsis, and cancer who do not have alcohol use disorder.^3^ ^,^ ^10^ ^,^ ^11^ ^,^ ^20^ Further testing in broader populations is needed to measure how age, gender, nutritional status, and other clinical factors interrelate with thiamine deficiency.

## CONCLUSION

In our unique study population of ED patients without a history of alcohol use disorder who had sepsis, cancer or diabetic ketoacidosis, independent risk factors for thiamine deficiency were advanced age, female gender, and leukopenia. Further research is indicated to define the epidemiology of thiamine deficiency in a broad cohort of adults with acute illness.
